# Process Analysis of PMMA Dental Waste Depolymerization in Semi-Batch Reactors

**DOI:** 10.3390/polym17192711

**Published:** 2025-10-09

**Authors:** Armando Costa Ferreira, Haroldo Jorge da Silva Ribeiro, Douglas Alberto Rocha de Castro, Marcelo Costa Santos, Caio Campos Ferreira, Fernanda Paula da Costa Assunção, Sérgio Duvoisin Jr., Luiz Eduardo Pizarro Borges, Nélio Teixeira Machado, Lucas Pinto Bernar

**Affiliations:** 1 Graduate Program of Natural Resources Engineering of Amazon, Campus Profissional-UFPA, Universidade Federal do Pará, Rua Augusto Corrêa No. 1, Belém 66075-110, Brazil; armandocostaferreira@hotmail.com (A.C.F.); ribeiroengq@hotmail.com (H.J.d.S.R.); caiocf7@hotmail.com (C.C.F.); 2 Centro Universitário Luterano de Manaus—CEULM/ULBRA, Avenida Carlos Drummond de Andrade No. 1460, Manaus 69077-730, Brazil; douglas.castro@ulbra.br; 3 Faculty of Chemical Engineering, Universidade Federal Rural da Amazônia—UFRA, Avenida Perimetral No. 2501, Belém 66077-830, Brazil; marcelo.santos@ufra.edu.br; 4 Graduate Program of Civil Engineering, Campus Profissional-UFPA, Universidade Federal do Pará, Rua Augusto Corrêa No. 1, Belém 66075-110, Brazil; fernanda.assuncao.itec@gmail.com; 5 Faculty of Chemical Engineering, Universidade do Estado do Amazonas-UEA, Avenida Darcy Vargas No. 1200, Manaus 69050-020, Brazil; sjunior@uea.edu.br; 6 Laboratory of Catalyst Preparation and Catalytic Cracking, Section of Chemical Engineering, Instituto Militar de Engenharia-IME, Praça General Tibúrcio No. 80, Rio de Janeiro 22290-270, Brazil; luiz@ime.eb.br; 7 Faculty of Chemical Engineering, Campus Profissional-UFPA, Universidade Federal do Pará, Rua Augusto Corrêa No. 1, Belém 66075-110, Brazil; machado@ufpa.br

**Keywords:** PMMA waste, process design, thermogravimetry, reaction mechanism, semi-batch reactors, depolymerization, pyrolysis, char formation, kinetics

## Abstract

This study examines the chemical recycling of polymethylmethacrylate (PMMA) dental waste in semi-batch fixed-bed reactors via pyrolysis, aiming to convert this waste into the valuable monomer methyl methacrylate (MMA). First, the effect of temperature is analyzed in a laboratory-scale (30 g) semi-batch reactor at 350, 400 and 450 °C. In order to visualize the combined effect of temperature and increase in bed volume, experiments conducted at 350 °C in the laboratory (30 g) and on a pilot scale (20 kg) are compared. Experiments conducted at 475°C on technical and pilot scales are also compared to elucidate this behavior. A detailed process analysis is presented, considering different experiments conducted in a semi-batch technical-scale reactor. Experiments were conducted in a 2 L reactor at temperatures of 425 °C, 450 °C and 475 °C to understand the effects of heating rate and temperature on product yield and composition. The results show that at 425 °C, MMA was the primary liquid component, with minimal by-products, suggesting that lower temperatures enhance monomer recovery. Higher temperatures, however, increased gas yields and reduced MMA yield due to intensified thermal cracking. This study also highlights that char formation and non-condensable gases increase with the reactor scale, indicating that heat transfer limitations can influence MMA purity and yield. These findings emphasize that for effective MMA recovery, lower temperatures and controlled heating rates are optimal, especially in larger reactors where heat transfer issues are more prominent. This research study contributes to scaling up PMMA recycling processes, supporting industrial applications to achieve efficient monomer recovery from waste.

## 1. Introduction

The chemical recycling of polymers refers to the transformation of polymeric waste into products useful to human society; it is the most prominent alternative for the treatment of plastic waste on a large scale [1]. Since the 1950s, world plastic production has increased exponentially from 2 million tons to over 450 million tons [2] due to the low production costs and technical possibilities displayed by polymers, as well as thanks to their range of material properties, such as chemical and mechanical resistance, ductility, hardness and others. In this context, plastic waste is a problem due to the same characteristics: the low cost of plastics facilitates their inadequate disposal, and characteristics like chemical and mechanical resistance reduce their degradability in the environment [3,4].

Only a small percentage of the plastics produced are recycled (9% each year), and their world production is increasing rapidly (9% each year) [2], turning the development and application of recycling strategies into a must. The main difficulties are related to obtaining and separating plastic waste and to the recycling process itself, which depends upon the type of waste recycled. The main strategies for the treatment of plastic waste are the following: disposal in landfills, energy recovery (incineration), mechanical recycling and chemical recycling [5].

Disposal in landfills should be avoided for plastics because they are not biodegradable, and incineration is a process demanding large investment and operational costs for the production of a resource (energy) readily available through other materials. The incineration of mixed plastic waste produces varied contaminants that must be treated on site, increasing processing costs [6].

The most commonly used process for recycling plastics is mechanical recycling, and it is usually applied to pre-treated waste (clean, decontaminated and dry) that has been partially or totally separated from other plastics, as in the case of the mechanical recycling of polyethylene (PE) and polypropylene (PP). The waste is milled and thermally molded (pressed or extruded) into new items. A maximum level of contamination is demanded, impeding the perpetual recycling of the waste and restricting its use in sanitary, food and health applications [1]. Some of the technical parameters of this waste, such as its mechanical resistance, brightness and opacity, are also reduced, reducing its application for engineering plastics such as acrylic polymers, made from methacrylic acid or polymethylmethacrylate (PMMA) [7].

For the complete recycling of polymers, chemical recycling must be used. Polymer molecules are converted into smaller ones through chemical reactions. Varied processes are described in the academic literature, including pyrolysis, gasification, hydrotreatment, viscosity breaking, and steam and catalytic cracking [8]. Ideally, the full depolymerization of the waste, i.e., its conversion into its monomer, is achieved; after purification, it can be used for the fabrication of completely new polymers.

PMMA is a transparent thermoplastic used as an engineering plastic. There are diverse applications for which some of its characteristics are demanded; these characteristics include mechanical resistance, non-toxicity, chemical resistance, transparency and many others [9,10,11,12]. It is used in acrylic lenses, dental cement, prosthetics, automobile parts and many more, with its annual production being estimated at more than 3.9 million tons [13]. PMMA can be recycled by pyrolysis, i.e., the thermal decomposition of the polymer in an oxygen-deficient atmosphere. The chemical recycling of PMMA is normally performed through fluidized bed pyrolysis, where PMMA particles are fluidized along inert particles (sand) by an inert gas (nitrogen or gases generated by the pyrolysis process itself). The heated particles and heated inert gas thermally decompose the polymer, generating over 90 wt.% of its liquid phase, containing over 90 wt.% of monomer methyl methacrylate (MMA) [7,14,15,16,17,18,19,20,21].

Pyrolysis in a fluidized bed is a mature process for the treatment of solid waste and its conversion into fuels and/or monomers, but it demands considerable investment costs with a complicated operation, and other reaction types and modes are researched [22,23,24,25,26,27]. Most of these are focused on using semi-batch reactors, where the initial feed is loaded previously inside the reactor but the formed vapors are allowed to flow freely out of the reactor. Research efforts make use of the simplest form of pyrolysis reactors with low investment and ease of operation. Table 1 comprises the main findings of these works and reveals that PMMA can be adequately depolymerized in semi-batch mode with liquid yields in the range of 50–99 wt.% and an MMA concentration higher than 75 wt.%. The biggest problems are related to the scaling-up of the process because of poor heat conduction in the fixed bed, increasing the decomposition of polymers instead of only their depolymerization into their monomers, augmenting gas yields and reducing MMA concentration [23,27].

The semi-batch fixed-bed reactor is of simple construction and operation, and it is of interest to analyze the problems concerned with its scaling-up process. Lab-scale reactors show that it is possible to obtain over 90 wt.% liquid phase with a high MMA concentration (over 90 wt.%). The poor heat transfer associated with larger fixed beds of polymer particles tends to increase rim temperature and reduce liquid-phase yields and MMA concentration due to further or side reactions. Also, one impending reason not to consider the use of semi-batch reactors, even on a small scale, is the existing dynamics of the process, which make it harder to analyze and optimize reactor conditions. This can be explained by the fact that at every given moment, the feed changes its composition with the reaction time, and the same can be said of its vapor products. Variables known to affect product composition and yields, such as temperature and heating rate, have to be carefully examined and evaluated by considering two factors: 1—the residence time of vapors; 2—the reaction time. As far as we know, there is no work available in the academic literature where this discussion is present.

In previous works, we described the process dynamics of PMMA depolymerization in a pilot reactor [24] and also the effect of increasing the process scale [27], but we did not analyze the effects of reaction time on product yields and composition. In this work, we conducted the depolymerization of PMMA waste in a technical-scale (2 L) fixed-bed semi-batch reactor and collected samples of the liquid phase according to the reaction time (time-fractioned samples) in order to evaluate how the process evolves dynamically. In this manner, it is thought that process variables and reactor design can be optimized for the depolymerization of PMMA, minimizing the intrinsic problems related to semi-batch reactors. Moreover, low and high temperatures of conversion are analyzed when considering different bed volumes (laboratory, technical and pilot scales), detailing further the combined effect of temperature associated with larger beds of heated polymer particles.

## 2. Materials and Methods

**Figure 1:**
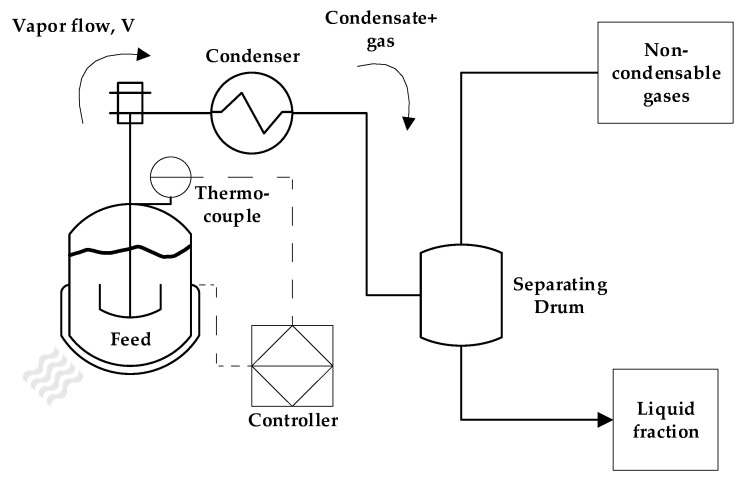
Process schematic of pyrolysis unit.

The dental waste was used as received from Dentisply Indústria e Comércio (Rio de Janeiro, Brazil), as milled irregular-shaped particles with a mean size of 9 mm. The dental waste PMMA received was composed of 95 wt.% of PMMA and 5 wt.% of EGDMA as a crosslinking agent. First, the process was investigated in a laboratory-scale reactor of 100 mL at different temperatures (350, 400 and 450 °C). Due to the endothermic nature of the PMMA depolymerization process and the semi-batch mode used, the processing temperature governs the extent of the reaction when the heating rate is fixed. Basically, most of the reaction happens at 350–400 °C, and the temperature is chosen based on the final temperature reached, controlling the reaction time and how much of the feed has been pyrolyzed and transformed into reaction products. Since it is expected that PMMA depolymerization generates little to no char product [28], the main reason to conduct the experiments on a lab scale was to observe which final reactor temperature is able to convert most of the feed into liquid and gaseous products. The laboratory pyrolysis unit is composed of a 100 mL cylindrical borosilicate-glass reactor inserted in a cylindrical electrically heated ceramic furnace of 1500 W. Reactor heating is controlled by a K-type thermocouple inserted between the reactor and the furnace wall connected to a PID controller. A heating rate of 10 °C/min was used here. The vapor outlet of the reactor is connected to a Liebig condenser, and the vapors are condensed with cooling water from a thermostatic bath operating at 25 °C. The non-condensable gases are vented from a glass separating funnel connected to the outlet of the condenser, and the liquid phase is collected from the bottom of the separating funnel. The schematic of the reactors on all process scales is shown in Figure 1.

The technical-scale pyrolysis unit was well described in previous works [29,30], and experiments were conducted in a 2 L semi-batch stainless-steel reactor heated by a refractory collar electrical heater of 3500 W insulated by a glass wool jacket. The vapor outlet of the reactor is connected to a stainless-steel tube condenser, refrigerated by a thermostatic bath using water as the cooling medium, and temperature is maintained at 15 °C. The condenser outlet is coupled to a cylindrical drum with upper and bottom outlets, used to separate the gas and liquid fractions, respectively. The liquid-phase samples are collected through the bottom outlet every 10 min of reaction until no more vapor or liquid condenses. The power used by the electrical heater is determined by a feedback controller located in the control panel of the unit, and it is based on the temperature measurement from a k-type thermocouple installed in a thermal well located in the pyrolysis reactor. This reactor also has an axial impeller for agitation and mixing, but in the case of solid particles of PMMA, it was not used.

A typical depolymerization experiment in a technical-scale reactor is performed by weighting 500–750 g of dental waste and loading it in the open reactor. The reactor is then coupled to its cover fixed on the pyrolysis unit framework. Eight sets of nuts and bolts and a graphite gasket are used to close the reactor and guarantee that no vapors leak during the experiment. The nuts and bolts are tightened in pairs diametrically opposed to each other, in order to guarantee the correct fixation of the reactor and no leaks. After that, the cooling and circulation of water is started in the cooling water thermostatic bath, and the circulation of water in the water seal of the mechanical impeller of the reactor is started.

Heating is performed in manual mode, where the temperature setpoint is chosen by the operator in the PID controller in order to obtain different heating rates. It was observed empirically that the power applied in the electrical heater is defined by the difference between the setpoint and the actual temperature in the reactor, and different heating modes are used depending on the error value (T_e_) between the setpoint and the actual reactor temperature. In the case of this controller, this value is set to 20 °C, supplying power to the heating element differently depending on this value:

1—T_e_ < 10 °C → The heating element provides power to increase the temperature slowly and maintain it once the reaction starts;

2—10 °C < T_e_ < 20 °C → The heating element provides power more frequently, increasing the reaction rate;

3—T_e_ > 20 °C → The heating element is always on.

In order to differentiate between the experiments, different setpoints for each different temperature (425, 450 and 475 °C) were used to obtain T_e_ of 10, 20 and 30 °C, respectively. For each experiment, the final temperatures were also different. It is possible to set the controller to automatic mode, where the heating rate is defined previously, but due to the weight of the stainless-steel reactor, there is a considerable lag between the setpoint and the actual temperature of the reactor, generating overshoots and high reaction rates. Also, the automatic mode does not take in consideration the presence of chemical reactions, especially endothermic ones, as in the case of depolymerization, increasing the error difference and generating high reaction rates.

As soon as vapors start to form, a timer is started, and the liquid phase is collected every 10 min of the reaction from the bottom tap of the separating drum. The time-fractioned samples are weighted and allowed to rest in a separating funnel in order to separate possible aqueous phases. They are then weighted again and conditioned in amber glass bottles.

Pilot-scale experiments were conducted in a 143 L semi-batch stainless-steel reactor previously described [24]. Two experiments were conducted at different temperatures (350 and 475 °C), in order to evaluate the process on a larger scale considering two extremes of depolymerization temperatures. The reactor was charged with 15–20 kg of scrapped dental waste for each experiment. The reaction is conducted in a similar way to the technical-scale reactor, where the setpoint is manually chosen by the operator in order to obtain a heating rate of 10 °C/min. When vapors start to form, liquid samples are drawn every 10 min of reaction.

It is important to note that all experiments (lab, technical and pilot) were conducted with no purge or inert gases flowing through the reactors, and the initial atmosphere was air. Oxygen was limited by sealing the reactors with their lids. The vapor formation when depolymerization starts ensures that no oxygen is admitted through the vapor outlet of the reactor.

GC-MS analysis was performed for each sample by diluting 1 µL of sample in 1 mL of acetone and injecting it into a gas chromatographer (Agilent Technologies, Santa Clara, CA, USA, Model CG-7890B) coupled to a quadrupole mass spectrometer (Agilent Technologies, Santa Clara, CA, USA, Model MS-5977A) in a capillary column of fused silica, SLBTM-5ms (30 m × 0.25 mm × 0.25 mm). The gathered spectra were compared with the NIST database, and no internal standard was used; the chemical composition was reported in area.% of chromatograms.

Physical–chemical properties were measured for the liquid phases obtained on a laboratory scale, since these samples were not fractioned according to the reaction time. Standard procedures of density (ASTM D4052 [31], 25 °C), kinematic viscosity (ASTM D445/D446, 40 °C) [32,33], acid value (AOCS Cd 3d-63) and refractive index (AOCS Cc 7-25) were used as described in previous works [24,27,29,30].

## 3. Results

### 3.1. Process Analysis of Laboratory-Scale Semi-Batch PMMA Depolymerization

The process parameters and product yields of the laboratory-scale depolymerization experiments are shown in Table 2. The lab-scale experiments were performed in order to observe the behavior of the depolymerization process in semi-batch mode but on a process scale where effects such as heat transfer could be neglected, with little effects on product yields and composition. It can be seen that all experiments presented similar reaction times (60–70 min) due to the similar weight of the feed present in all experiments (30–40 g), showing that the effective heat used to complete the depolymerization was similar in all of them. In the experiment where 350 °C was used as the final temperature, it can be observed that the depolymerization reaction tends to stall when producing liquid-phase monomer and starts to produce more gas-phase products, increasing gas yields and leaving a charred residue in the reactor, along with unreacted feed. The mechanism of PMMA depolymerization depends upon some properties of the feed combined with process variables such as temperature, heating rate and reactor geometry [7,14,15,16,17,18]. 

It is known in the academic literature that PMMA fabricated via a free-radical polymerization mechanism (the most common) decomposes via chain-end initiation and/or mid-chain random scission and depropagates to the monomer until a termination reaction occurs via disproportionation, recombination or chain transfer to the solvent [34,35,36,37,38]. The average number of depropagation events between initiation and termination events is called the zip length, and it depends on the ratio of initiation and termination to propagation reaction rate constants and can be expressed as the probability p of a depropagation event over a termination event after initiation [34], as defined by Equation (1). The zip length (1/ε) can be expressed in terms of this probability, as shown in Equation (2).

(1) $$p=k_{d}\left(\frac{2k_{i}}{k_{tr}k_{t}{\left[R\right]}^{2}}\right)\;\;\;\;$$

(2) $$\frac{1}{\varepsilon }=1+p\left(1+\frac{k_{t}}{k_{d}}p\right)\;\;\;\;$$

**Figure 2:**
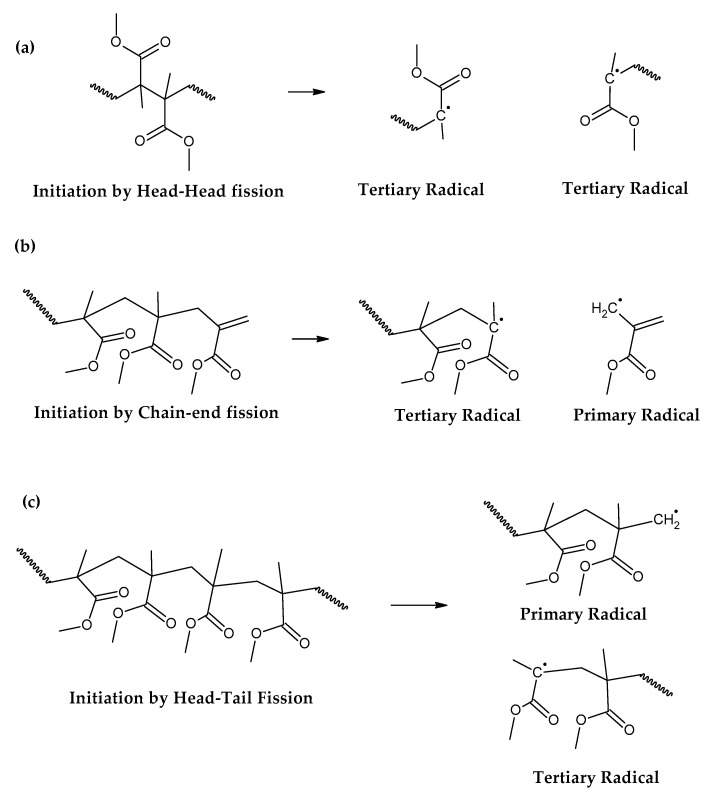
Initiation reactions of PMMA depolymerization. (**a**) Initiation by head-head fission. (**b**) Initiation by chain-end fission. (**c**) Initiation by head-tail fission.

**Figure 3:**
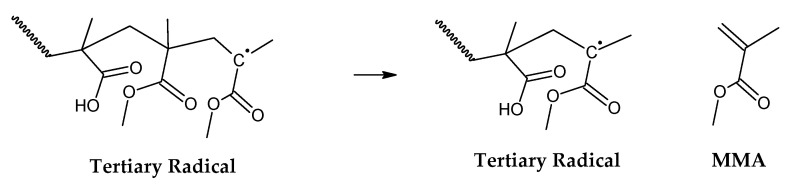
Depropagation to the monomer MMA.

**Figure 4:**
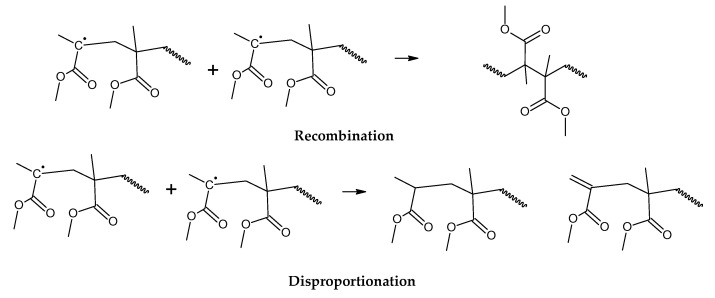
Termination of depolymerization reactions of PMMA.

For depolymerization reactions where the zip length is larger than the degree of polymerization, the polymer chain reacts fully, leaving no smaller species behind and consequently no char. If the zip length is lower, then a longer polymer residue is left behind. Depending on the dispersity of molecular mass of the reacting polymer and the type of termination (disproportionation or recombination), this residue may be collected in solid, liquid or gas phase [34,36]. But this mechanism does not take into account competitive side reactions, such as polymer charring in the case of thermal decomposition of PMMA. The overall reaction represents a complex sequence of reactions occurring during high-temperature pyrolysis of PMMA, generating solid carbon and gases such as aromatic hydrocarbons, CO, CO_2_ and H_2._ These reactions occur between pyrolysis products, mainly through aromatization and condensation reactions but also gasification and reduction reactions in high-temperature zones. To some extent, they may occur with any carbonaceous feedstock in the reactor [39,40,41,42]. This phenomenon changes the composition of the polymeric chain during semi-batch depolymerization and virtually stops the unzipping and depropagation to the monomer when a certain temperature is reached. This could explain why some polymers tend to stop the depolymerization reaction before even the monomer equilibrium concentration, i.e., the point where propagation and depropagation reactions reach the same reaction rate [34]. The mechanism of thermal decomposition of PMMA, composed of initiation, depropagation and termination reactions, is shown in Figure 2, Figure 3 and Figure 4, respectively. It is important to note that termination of reaction via chain transfer to the solvent is not possible during thermal depolymerization of PMMA in solid phase, and it is not shown among the mechanisms in Figure 4.

**Figure 5:**
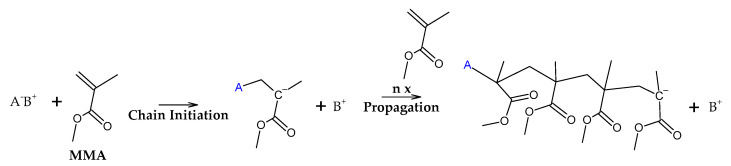
Anionic polymerization mechanism of PMMA.

The initiation reactions are shown according to increasing bond energy. A head–head linkage, shown as (a) in Figure 2, is the lower in bond energy, and it is the most likely to break during thermal decomposition. It is formed by termination by recombination of the polymer chain, and it happens 20% of the time during FRP polymerization [42]. FRP-made polymers degrade at lower temperatures (140–200 °C), but this type of termination is not present in anionic polymerized PMMA, where propagation occurs almost exclusively by H-T propagation, termination only occurs deliberately and no H-H linkages are formed [42]. The mechanism of anionic polymerization is shown in Figure 5. The second most likely bond cleavage is at vinyl chain-ends, formed during termination by disproportionation, also not present in anionic polymerization. This type of initiation (Figure 2b) usually occurs around 300-323 °C, lower than for random H-T fission (327–402 °C) (Figure 2c), where usually, most of the decomposition is observed in mass loss in thermogravimetry experiments.

Conducting PMMA depolymerization at 350 °C does not supply sufficient heat to conduct the endothermic cleavage and depropagation of the polymer chain to the monomer for all the feed, the effective zip length of depolymerization is lower than the polymerization degree, and the reaction terminates with intermediary products, which could serve as reagents for other reactions, such as hydrogen abstraction events, condensation and aromatization, and formation of polyclyclic aromatic hydrocarbons, char and gases. This is why the experiment conducted at 350 °C displayed lower liquid-phase yields (~49 wt.%), where the distilled formed monomer is encountered, and increased solid and gas yields, ~39 wt.% and ~12 wt.%, respectively. Low temperatures of depolymerization favor charring and other types of reaction due to random-chain scission being suppressed by the low heat supplied for this endothermic reaction. As the reaction times were similar among all experiments, the product distribution changes from the complete depolymerization of the PMMA chains to a mix of depolymerization and charring reactions. This is clearly shown by the increased gas yield obtained, showing that 60 min of reaction time is sufficient to conduct the reaction, since the reaction is stopped only when no more liquid is collected in the separating funnel, indicating that there is no more depropagation of the polymer chain. The results were similar to those obtained by Braido et al., who conducted PMMA dental waste depolymerization at 350 °C for 1.5 h in a semi-batch glass reactor of 424 mL and obtained ~30 wt.%, ~60 wt.% and 10 wt.% solid, liquid and gas phases, respectively [23].

Higher temperatures of depolymerization (400 and 450 °C), on the other hand, could reach the temperature needed for the almost complete depolymerization of PMMA waste, since random scission is favored at temperatures of 327–402 °C, culminating in the chain depropagation of the entire polymer to the monomer. It can be seen that the charring and gas-forming reaction still happens to some extent and competes with depropagation, as indicated by the solid yield of 6.49 wt.% in the 400 °C experiment. Looking at the solid yield for the experiment at 450 °C, 0.68 wt.%, one could think that the charring reactions can be suppressed by high temperatures of depolymerization, but this is only partially achievable in semi-batch reactors, where the formed vapors are rapidly removed from the heated bed. Since heat is being directed to the endothermic cleavage of chemical bonds and the vaporization of the formed products, supplying more heat fuels more cleavage and more vaporization, and the temperature tends to stall around a certain value (or even decrease) when the reaction is at full speed. In the case of PMMA dental waste, this plateau is around the temperature of random scission (327–405 °C), so temperatures higher than 400 °C are only achievable when a large portion of the feed has already been vaporized and collected in the separating funnel. This is shown by the similar liquid-phase yields at 400 and 450 °C, ~91 wt.% and ~95 wt.%, and the higher quantity of gases at 450 °C, 4.58 wt.%, indicating that the increased liquid and gas yields are derived from the pyrolysis of the formed char in the reactor. Additionally, the GC-MS analysis of the liquid phase on technical and pilot scales showed that for high reaction times, i.e., high temperatures (>400 °C), side products different from liquid MMA are observed, such as aromatic hydrocarbons, formed from the charring of the polymer feed. Unfortunately, due to the small volume of liquid phase obtained in the laboratory-scale experiments, no fractionation of the liquid phase was performed according to the reaction time, all the product was collected in a single flask, and the difference in liquid phases could not be observed for its measured physical–chemical properties, which are shown in Table 3.

It can be seen that the liquid phases from all experiments were similar to each other, indicating that the majority of compounds present were the same for all of them. Indeed, depolymerization of PMMA polymer usually produces a liquid phase rich in MMA monomer [7,14,15,16,17,18]. Side products are formed in much lower quantities and, as we detailed before, tend to distribute themselves in solid and gas phases. Only a minority of liquid side products are formed at high reaction times, and their quantity are not sufficient to alter the measured physical–chemical properties. This can be observed when considering the acid value of the liquid phases, since purified liquid MMA does not present considerable acidity (it presents traces of acidity in the form of methacrylic acid); this parameter is indicative of the presence of contaminants in the liquid phase, and all experiments presented similar values. The results revealed that the liquid phase of PMMA depolymerization presents similar values to the liquid monomer, with its properties also presented in Table 2, indicating that the liquid product is rich in MMA. It is important to note that even though the physical–chemical properties are similar, the lab-scale liquid product of PMMA depolymerization presented a dark color, and further purification is needed in the form of fractional distillation, as suggested by Achilias et al. [22]. The increased acid value and reduced kinematic viscosity of the liquid-phase indicates the presence of contaminants that could affect the process of repolymerization. Braido et al. achieved moderate repolymerization after conducting fractional distillation of the liquid product of PMMA depolymerization [23]. In order to achieve better polymerization, a second step of distillation or increased reflux ratio should yield purified product capable of complete repolymerization similar to the virgin monomer.

Questions remain about the nature of the contaminants present in the liquid product of PMMA depolymerization. A well-known contaminant in pyrolysis oils is graphitic carbon formed during pyrolysis, which is divided in fine particles, is carried away during pyrolysis and ends up with the condensed liquids. It is possible to remove this carbon by adsorption and filtration with activated carbon with an adequate specific surface and porosity. Normally, most pyrolysis oils change from dark to a brown color, and we can assume that the removed carbon has little effect on the acid value and viscosity of the liquid product of PMMA depolymerization. The nature of the contaminants affecting these properties will be discussed in Section 3.3.

### 3.2. Process Analysis of Technical-Scale PMMA Depolymerization

Table 4 presents the main findings in respect of the product yields obtained by depolymerization considering different temperatures (425, 450 and 475 °C) for the technical-scale reactor of 2L. It can be seen that liquid-phase yields tend to decrease with the increase in temperature, the gas phase yields tend to increase, and the solid phase tends to decrease until a minimum is reached. Similar findings are reported in the academic literature [22,23,24,26,27]. Achilias et al. reported that increasing the temperature above 450 °C increased gas yields and lowered liquid yields [22]. Braido et al. found only a minor increase in gas yields when polymerizing crosslinked PMMA from dental waste when increasing the process temperature [23]. The presence of crosslinking in PMMA is said to increase gas formation and unwanted carbon [17]; Braido and collaborators reported that crosslinked PMMA did not melt during pyrolysis, while the homopolymer tested did, but they attributed the small increase in gas yields with the process temperature to the excellent properties of heat transfer of the small-scale reactor. In the larger-scale technical unit, a considerable amount of char (~25 wt.%) and reduced liquid yields (~65 wt.%) were obtained compared with the laboratory-scale reaction (~90 wt.% liquid yield) [23]. Santos et al. [24] pyrolyzed dental waste in a 143 L pilot-scale fixed-bed reactor; they reported that the liquid yield shows a first-order decay function with temperature and found increased gas yields (~30 wt.%) compared with the results obtained by Braido et al. on a technical scale (~6 wt.% gas yields). The char yields obtained by Santos et al. were in the range of 11–14 wt.%, lower than Braido et al.’s but higher than the ones obtained in this work with a technical-scale unit, where higher temperatures were used for conversion and a char yield of ~2–3 wt.% was obtained for temperatures higher than 425 °C. Braido et al. commented that the depolymerization was not complete on a technical scale after 1 h of reaction and a higher number of impurities was found in the reactor on a technical scale compared with the lab-scale reactor, indicating that higher temperatures favor the charring (polymerization to higher-molecular-weight hydrocarbons) of the crosslinked polymer; even though it is possible to increase the liquid yield, it is not from the monomer MMA but from higher-molecular-weight products, such as aromatic hydrocarbons. This is not observed on a lab scale, where heat transfer problems are negligible but become considerable when increasing the process scale. The formed char can be further pyrolyzed by increasing the process temperature, generating products in the gas phase, as it is observed in this work, where higher temperatures of depolymerization (425–475 °C) were investigated compared with Braido et al. (400 °C) [23]. This effect is further augmented when a larger fixed bed is used, as it was shown by Santos et al.’s study, where large gas quantities and lower char yields were obtained at lower temperatures (345–420 °C) [24]. Poudel et al. pyrolyzed artificial marble powder composed of filled PMMA and reported that increased temperature reduces the recovery of MMA from liquid phase due to the presence of impurities, even though an increase in liquid yield occurs with temperature, pointing to the necessity of homogeneous temperature distribution in the reactor for the maximization of the recovery of the monomer [26]. For fluidized bed reactors, where the heat transfer between the heated gas phase and each individual particle is fluidized, this effect is minimized, and a high-MMA-containing liquid phase is obtained [7,14,15,16,17,18].

**Figure 6:**
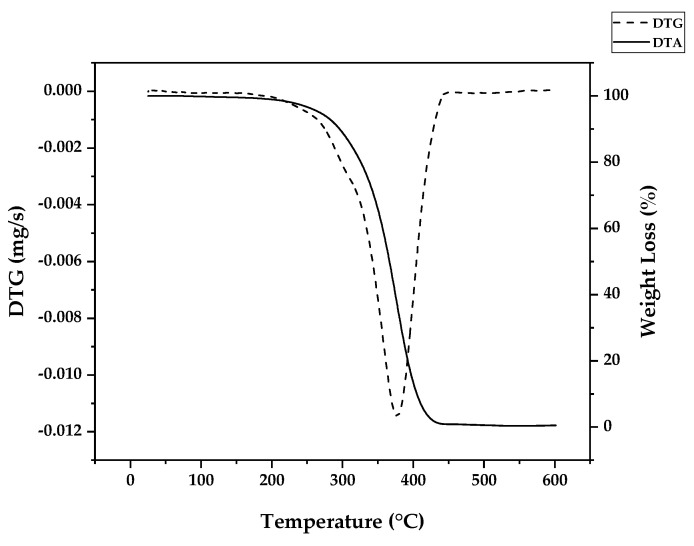
TGA/DTG profile of PMMA dental waste.

Independently of the type of polymerization used to fabricate the polymer, due to the different energies present in different linkages, it is possible to obtain an approximate proportion of linkage types in determined polymers by thermogravimetry experiments, where a controlled mass of a polymer is degraded at different temperatures at an inert atmosphere and the mass loss (or its derivative) at each respective temperature of decomposition (TGA/DTG) is observed. Due to the small mass used (~5 mg), heat transfer and diffusion effects on thermal decomposition were negligible, and chemical effects were maximized; i.e., the parameters observed are largely due to linkages and substances present in the sample [43]. Figure 6 shows the weight loss of PMMA dental waste obtained by conducting a TGA/DTG experiment using a 10 °C/min heating rate, nitrogen atmosphere and a maximum temperature of 600 °C. The DTG curve shows a peak of maximum decomposition at a temperature of 376 °C, indicating that most of the thermal degradation happens because of random H-T fission. Indeed, for FRP or anionic polymerization, most of the bonds in high-molecular-weight polymer chains are H-T linkages. But, from the weight loss curve, one can observe that thermal degradation started at as early as 300 °C, pointing to the presence of vinyl chain-ends formed due to disproportionation in FRP, supposedly not present for anionic polymerization. Nevertheless, no decomposition was observed below this temperature, indicating that no H-H linkages were present due to termination by recombination, supposedly happening around 20% of the time with FRP. This effect could be suppressed by the presence of the crosslinking agent EGDMA, giving thermal stability to the linear chain of the polymer [35].

**Figure 7:**
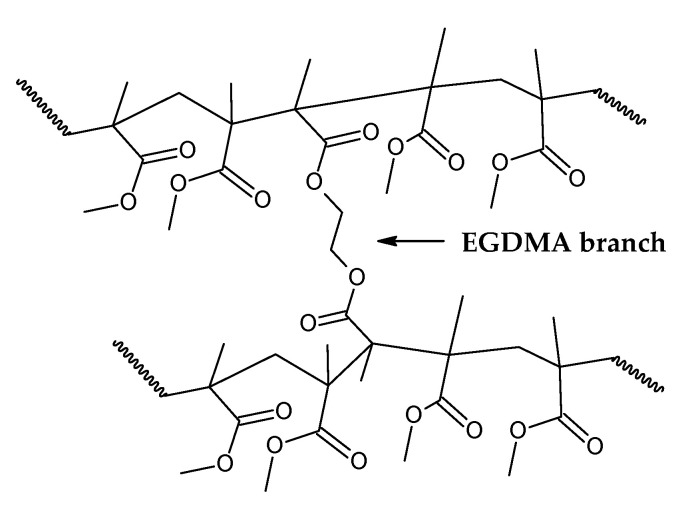
Branched chain of EGDMA-PMMA polymer.

Da Ros et al. [35] modeled the kinetics of thermal depolymerization of PMMA dental waste containing 5 wt.% EGDMA as the crosslinking agent. They compared thermograms with pure PMMA and concluded that EGDMA-crosslinked PMMA degrades mainly through random H-T fission, suppressing early degradation by H-H fission. While pure PMMA showed DTG curves with three clearly distinct peaks, for EGDMA-PMMA, only two peaks of degradation were observed, a small peak at 250–300 °C (vinyl chain-end fission) and a much higher peak at 376 °C (random H-T fission), and the kinetics of depolymerization could be modeled using a consecutive two-step reaction, dominated by the random H-T fission mechanism. This correlates well with the necessity of a high degree of polymerization, i.e., a high-molecular-weight polymer, in dental cement to obtain a polymer with the desired mechanical properties [44,45,46]. In such a polymer, most of the bonds in the branched polymer chain are H-T bonds formed during monomer propagation. In fact, Da Ros et al. could model the depolymerization kinetics even when considering a one-step reaction due to dominance of random scission in the mechanism [35]. When observing the thermogram in Figure 6 (in this work), one can observe that for this dental waste cement, only a clear, distinct peak was observed, but thermal degradation of 22 wt.% was observed before a temperature of 330 °C was reached (temperature of vinyl chain-ends fission), indicating that most termination reactions in the FRP process of crosslinked PMMA were due to disproportionation. It seems that the branching of the polymer chain minimizes the formation of H-H linkages due to recombination reactions (or at least suppress its early breaking in thermal decomposition) and allows only 22% of the polymerization reactions to terminate, while 78 % of reactions continue until all polymers are consumed in polymerization, approximating the process of depolymerization of FRP PMMA to anionic polymerized PMMA and conferring thermal stability to the polymer. This makes it possible for the branched chain of PMMA to decompose through the depropagation mechanism (the unzipping of the polymer chain) until all the polymer is converted into monomers. Since most of the depolymerization occurs due to random fission, it is speculated that FRP combined with crosslinking minimizes the formation of chain-end groups. Figure 7 shows how EGDMA inserts itself and creates an increased-elasticity and -strength PMMA polymer [44,45,46].

Indeed, the branching of the polymer chain increases its molecular weight and improves properties such as low solubility in organic solvents and improved strength and elasticity [45]. As it was suggested by the thermal analysis and application of PMMA as dental cement, a high degree of polymerization is expected for this polymer, since most of the decomposition happens due to H-T fission of the polymeric chain, indicating a high polymerization degree. This parameter (Equation (3)) is the number of monomer units in a polymer or oligomer molecule, and it can be represented by the ratio of the number-average molecular weight of the polymer (${\bar{M}}_{n}$) to the molecular weight of the monomer unit (M_0_). Dental resins are usually prepared by FRP of prepolymerized PMMA beads with benzoyl peroxide as the initiator and liquid MMA as the monomer. The prepolymerized beads usually present high molecular weight (120,000–996,000), which is further increased after repolymerization [45]. Since the molecular weight of MMA is 100.177 g/mol, dental resins usually present degrees of polymerization higher than 120. For saturated chains of PMMA polymer, the degradation rate (mass loss) by random scission is described by Equations (4) and (5) for zip lengths higher and lower than the degree of polymerization, respectively. K_s_ is the rate constant for random-chain scission (H-T fission), and γ is the reciprocal of the average zip length between initiation and termination reactions [47].

(3) $$DP=\frac{{\bar{M}}_{n}}{M_{0}}\;\;\;\;$$

(4) $$\frac{d[M]}{dt}=-k_{s}DP\left[M\right];zip\;length\gg DP\;\;\;\;$$

(5) $$\frac{d[M]}{dt}=-2k_{s}\left(\frac{1}{\gamma }\right)\left[M\right];zip\;length\ll DP\;\;\;\;$$

If the zip length is lower than the degree of polymerization, it means that the depropagation of the polymer into the monomer does not happen to completion, i.e., it terminates by forming intermediary products of depolymerization. The observation of the thermogram from Figure 6 reveals that virtually all PMMA dental waste is converted into gases (99.5% of weight loss), suggesting that even if the zip length of this depolymerization process is lower than DP, all terminated products are converted into gases below a temperature of 450 °C. If the zip length is lower than DP, the vapor composition should change from an enriched-MMA vapor to the pyrolysis products of the terminated products of the depolymerization reaction at later stages (higher temperatures). Beyond that, there is also the possibility of side reactions between depolymerization products [37] and charring reactions [39,40,41,42].

**Figure 8:**
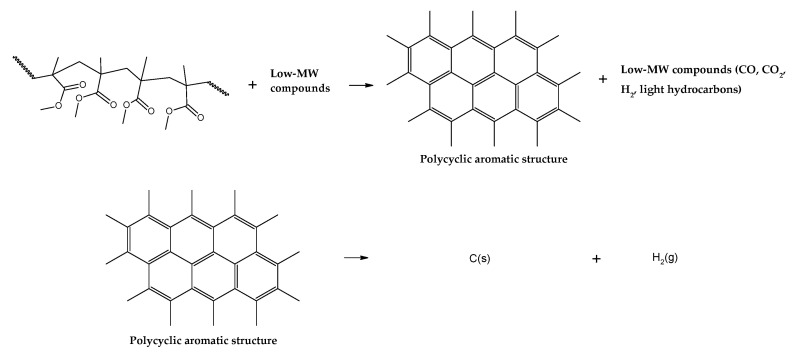
Charring reactions of PMMA.

When one compares the results obtained in the thermal analysis with the yields of the products of the depolymerization of PMMA dental waste in the technical unit of IME, some char is formed in the larger-scale unit (~2 wt.%), indicating that the overall depolymerization mechanism includes more reactions than what it is initially supposed for PMMA thermal degradation. Indeed, mechanisms of depolymerization are usually kinetically controlled and depend upon heat transfer and diffusion coefficients, being influenced by sample thickness [37]. This means that other reactions could be involved and be determinant in final product distribution and composition. Experimentation with the pyrolysis of carbonaceous materials usually results in higher char yields when using low temperature and heating rates [39,40,41,42]. Although not fully known, it is estimated that char is formed due to inter- and intramolecular rearrangement reactions, forming a polycyclic aromatic structure, as shown in Figure 8. Further reactions include hydrogen abstraction events, forming hydrogen gas and graphitic carbon. Due to the lower actual heating rate in the polymeric bed in the technical-scale reactor, more char is formed than in the small-scale thermal reactor of TGA analysis.

**Figure 9:**
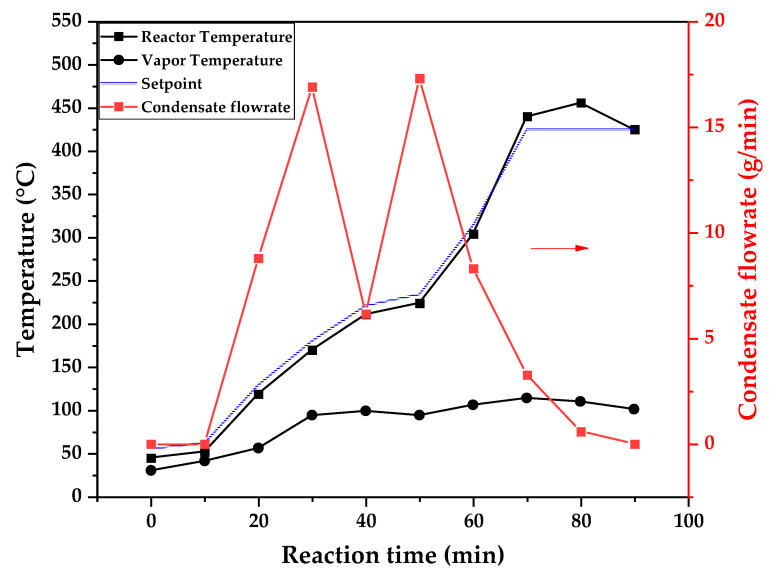
Temperature profile of PMMA dental waste depolymerization at 425 °C.

**Figure 10:**
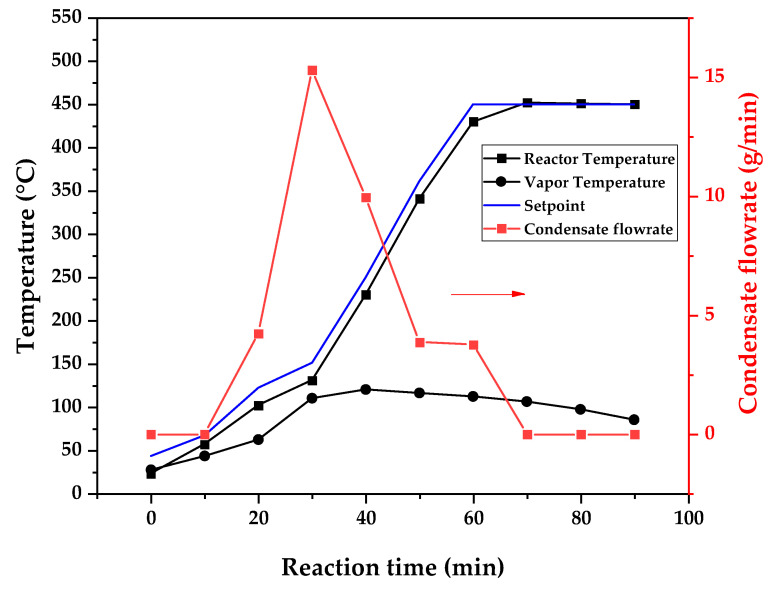
Temperature profile of PMMA dental waste depolymerization at 450 °C.

**Figure 11:**
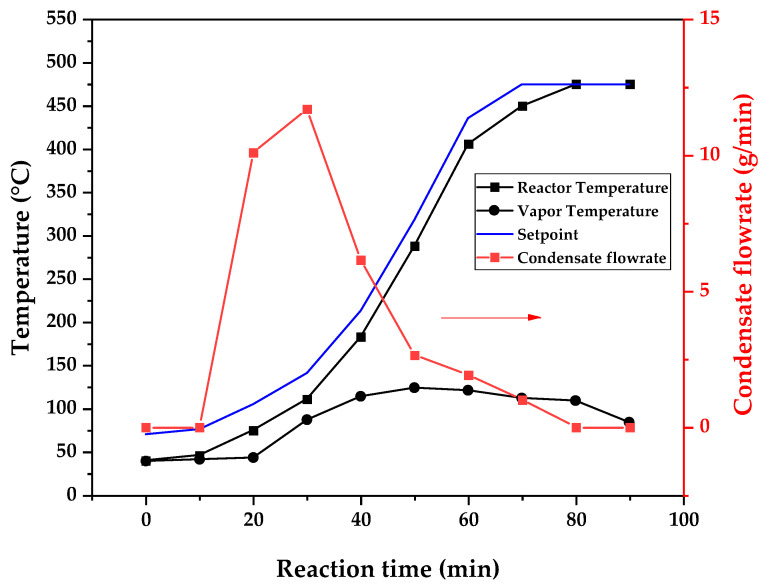
Temperature profile of PMMA dental waste depolymerization at 475 °C.

Figure 9, Figure 10 and Figure 11 show the temperature profiles of the reactor and vapor temperature according to the reaction time for PMMA dental waste depolymerization at 425, 450 and 475 °C, respectively. In order to effectively analyze the semi-batch depolymerization process, the vapor flow formed during the heated depolymerization reaction for each temperature is also included in the graphs of Figure 9, Figure 10 and Figure 11. The condensate flowrate was calculated by collecting the liquid condensate at each timestamp (every 10 min) and weighting it. The flowrate was then calculated by dividing the amount collected by the amount of time passed (10 min). Although presenting high variability, it is mainly shown in order to visualize the relation between reaction temperature and semi-batch experiment conduction, i.e., the temperature where the reaction starts and finishes. The condensate flowrate is shown in Figure 9, Figure 10 and Figure 11 as a double axis on the right. In order to improve visualization, the condensate flowrate line and its axis are shown in red color. It can be observed that vapors started to form and condense at considerably lower temperatures than what was observed in the thermal analysis in Figure 6, where thermal degradation (weight loss) started above 200 °C and, for the technical-scale unit, vapors started to form and condense in the liquid state at 75 °C. We believe it to be an effect of the greater dimensions of the bed of polymer particles inside the technical reactor compared with the thermobalance of the TGA analysis. Since PMMA dental waste presents poor heat transfer properties, a larger fixed bed of polymers creates a temperature gradient between the rim and center of the reactor; i.e., when the center presents a certain temperature, it is expected that the rims of the fixed bed are at higher temperatures. Since the reactor thermocouple is located at the center of the reactor, it probably showed lower temperatures at the reaction’s beginning, stabilizing at a correct value at higher reaction times. Ribeiro et al. conducted PMMA depolymerization on different production scales and concluded that this effect is augmented for larger semi-batch reactors [27]. By observing Table 1, one can perceive that it also affects the product distribution in different phases. Even though it is possible to obtain high liquid-phase yields in lab-scale (30 g) PMMA dental waste depolymerization [22,23], only 81 wt.% liquid-phase yield could be obtained at a temperature of 425 °C in the technical-scale unit, and at higher temperatures (450 and 475 °C), this yield was much lower, around 62 wt.%.

This behavior seems correlated to the different regimes of heating used for each experiment. Even though the temperature profiles of the three experiments were alike, including the reaction times, the condensate flowrates decreased with the temperature increase due to the formation of gaseous non-condensable substances, reducing the liquid-phase yield and increasing the gaseous yield, as shown in Table 1. It can be observed that for endothermic reactions being conducted in semi-batch reactors, almost no temperature increase occurs when an endothermic process is in place; in fact, added energy is used to increase the rate of the endothermic processes, such as the depolymerization reaction and the vaporization of the formed products [29,30]. Only after these processes are completed, the temperature starts to rise again to the desired setpoint. In fact, the different temperatures used in the experiments actually represent different heating rates and final temperatures of reaction. In Figure 9, Figure 10 and Figure 11, the setpoint curve represents the temperature chosen by the operator to indirectly set the heating rate used. This is performed by choosing setpoints with different temperature errors from the actual reactor temperature, 10, 20 and 30 °C for the experiments at 425, 450 and 475 °C, respectively. This was performed in order to achieve different profiles of heating from the heating element providing the driving force of the chemical reaction. From the relay indication of the control panel, it is possible to visualize if power is supplied to the heating element. The amount of power of the heating element is controlled by the amount of time during which it receives electrical current and, due to the configuration of the controller, the heating element is set to operate at different levels depending on the temperature error difference (T_e_) between the setpoint and the actual reactor temperature. For the technical-scale reactor, this error difference was set to 20 °C, causing it to operate in three different modes, as detailed in Section 2.

In Figure 9, Figure 10 and Figure 11, the liquid condensate flowrates decreased with the increase in processing temperature, but the actual flowrate of vapors probably increased due to the formation of more non-condensable gases. The increased heating rate was able to further react the MMA formed during PMMA dental waste depropagation into different reaction products, mostly in the gas phase, probably in the form of carbon dioxide and light hydrocarbons. Further testing of the composition of the gas phase is needed in order to obtain detailed characteristics of these secondary reactions.

### 3.3. Chemical Composition Analysis

**Figure 12:**
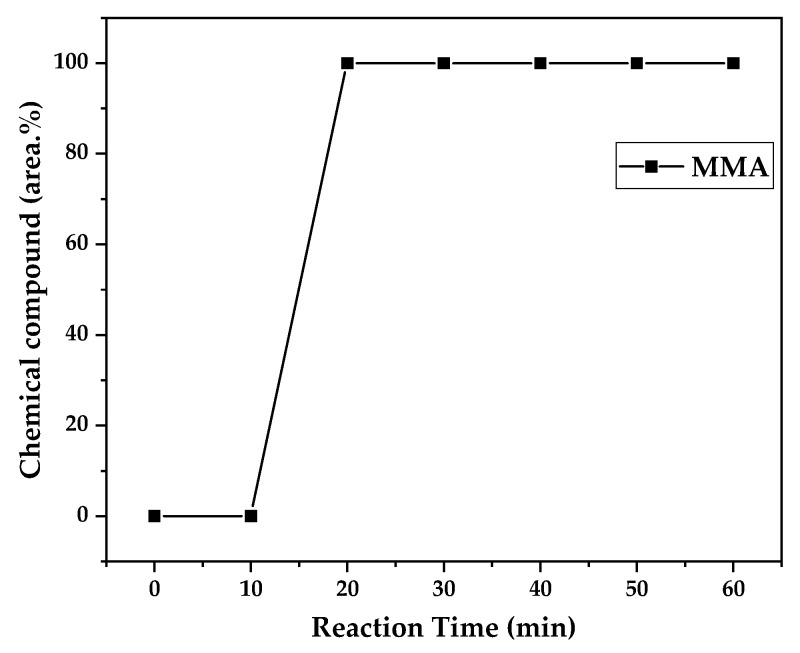
Chemical composition according to reaction time of liquid product of PMMA dental waste depolymerization at 425 °C.

**Figure 13:**
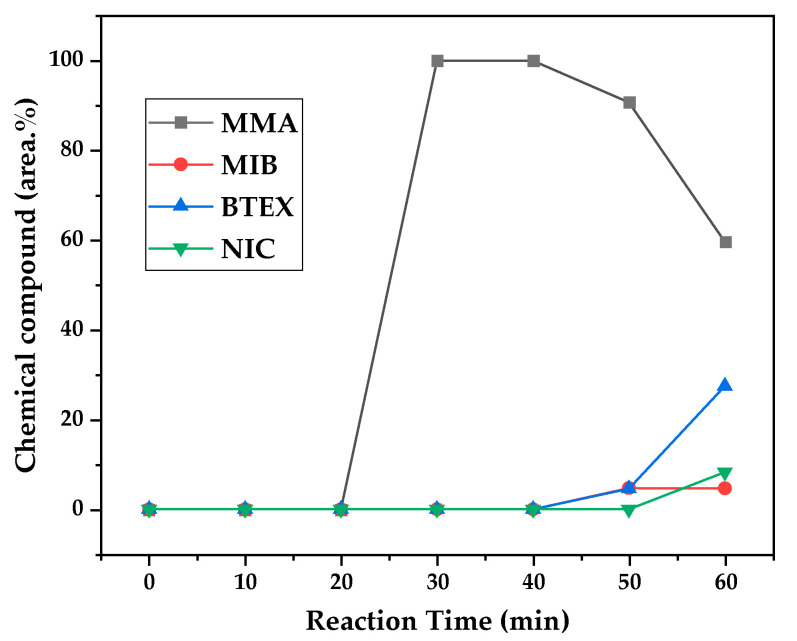
Chemical composition according to reaction time of liquid product of PMMA dental waste depolymerization at 450 °C.

**Figure 14:**
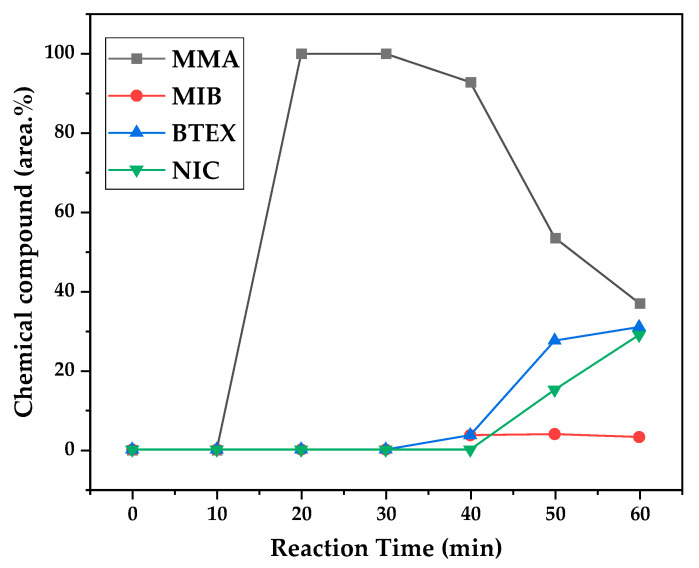
Chemical composition according to reaction time of liquid product of PMMA dental waste depolymerization at 475 °C.

Figure 12, Figure 13 and Figure 14 present graphs showing the how chemical composition of the liquid product formed during PMMA dental waste depolymerization changed over reaction time at the three temperatures of depolymerization. Initially, the liquid product is composed by the monomer MMA, being the only product of depolymerization at low temperature and reaction times, corroborating the established depolymerization mechanisms of PMMA, where most of the vapor product formed is due to the depropagation reaction, producing the monomer MMA. As the reaction proceeds, since it is a semi-batch reactor, the degree of polymerization of the remaining feed in the reactor decreases over time but so does the zip length due to the diminishing length of the polymer chain. Additionally, the polymer chains are also transformed into other products due to condensation and aromatization reactions, turning into char and other cracking products of char pyrolysis. Higher heating rates can also increase or accelerate some side reactions, increasing product complexity and distribution. These reactions compete with the depropagation of the polymer chain into monomers, and eventually other products are formed in the reactor and vaporize, condensing into the liquid product of depolymerization. This can be seen in Figure 13 and Figure 14 for the reactions conducted at 450 and 475 °C, respectively. After 20 min of liquid condensation, the composition starts to change from 100 area.% of MMA to side products as methyl isobutyrate (MIB); aromatic hydrocarbons such as benzene, toluene, and xylene (BTEX); and other complex compounds that could not be accurately identified, supposedly hydrocarbons and oxygenated compounds with long chains of carbons (char and tar precursors) [39].

As it was seen in the previous section, the mechanism of depolymerization is affected by the heating rate used, and it is related to the final temperature of reaction in the technical-scale unit. By observing the temperature profiles in Figure 10 and Figure 11, higher heating rates produced lower liquid condensate yields, but the chemical composition of the obtained liquid fraction shows that for most of the time, it was majorly composed of MMA, indicating that initially, most of the secondary products formed were gases, being in the non-condensable gases phase, and were formed due to secondary cracking reactions of the diffusing MMA through the heated polymeric bed. For the experiment conducted at 425 °C, the liquid product was composed of 100 area.% of MMA, and no side products were formed in the liquid phase with a char yield of 9.44 wt.%. For the experiments using higher heating rates and final temperatures in Figure 13 and Figure 14 (450 and 475 °C), lower char yields (~2.5 wt.%) but an increased presence of side products were obtained, showing that both the final temperature and heating rate influence the formation of char and side products.

The presence of these side products in the vapor composition of PMMA dental waste depolymerization can also be visualized by the vapor temperatures shown in Figure 9, Figure 10 and Figure 11. It is possible to observe that the vapor temperature tends to maintain its value near the boiling point of the vaporized mixture of substances coming out of the heated reactor. Since most of the liquid condensate is MMA, values around 101 °C should be observed (boiling point of MMA). For the experiment in Figure 9 (425 °C), the maximum vapor temperature was 114 °C (70 min of reaction), but most of the liquid was formed below 100 °C. For Figure 10 (450 °C) and Figure 11 (475 °C), the vapor temperature increased to over 110 °C at 30 and 40 min of reaction, respectively and the maximum temperatures were 120 and 124 °C. The chemical compositions in Figure 13 and Figure 14 reveal that the vapor temperature increased due to the presence of side products in the liquid condensate, formed after 30 min of reaction.

The mechanism of depolymerization is also affected by the existing kinetics in the reactor, i.e., the heat transfer and diffusion of formed products through the polymer particle’s bed. In small-scale and miniaturized reactors (such as the thermobalance of TGA/DTG analysis), the geometry of the particle’s bed is small compared with the heat transfer and diffusion needed. As the size of the reactor increases, the effect of heat transfer and diffusion also increases, changing reaction rates and product composition [27]. Since the temperature controller thermocouple is located in a thermal well near the center of the particle bed, there is probably a temperature gradient from the rim to the center of the cylindrical reactor, meaning that the actual temperature at the rims should be higher than the setpoint. This can explain why semi-batch fixed-bed reactors produce more gas and less liquid-phase yields compared with small-scale semi-batch reactors. The higher temperature at the rim can further crack the products of depolymerization, forming non-condensable gases such as CO, CO_2_, H_2_ and light hydrocarbons (CH_4_, C_2_H_2_ and others), increasing gas yields and reducing the actual recovery of MMA. Since formed products are part of the gas phase, there is no change in the chemical composition of the liquid phase until most of the feed converts into char and the char pyrolysis dominates product distribution and composition.

By observing the chemical composition in Figure 14, it is possible to observe that the main contaminants in the liquid product are BTEX compounds, especially toluene. This does not explain why there was a reduction in kinematic viscosity (Table 2) for the liquid product of depolymerization, because toluene presents a higher value of kinematic viscosity than MMA. It does not explain why the acid value was ~2.0 mg KOH/g, since toluene is not an acid substance. We believe it to be due to the non-identified compounds, whose amounts start to be considerable near the end of reaction, along with BTEX compounds. Better separation needs to be conducted in order to identify the actual contaminants, since they seem to be aromatic compounds of high molecular weight. The sharp peak of MMA in the sample makes it difficult to distinguish other substances present, and maybe, the purification of the sample is needed in order to better analyze the minority compounds.

### 3.4. Combined Effect of High-Temperature Depolymerization and Large Beds of PMMA Particles

In order to better visualize the effect of high-heating rates and high temperatures of PMMA dental waste depolymerization when considering the semi-batch mode, Table 5 compares the product yields obtained in PMMA depolymerization at 350 °C on laboratory and pilot scales with the experiments performed at a higher temperature of 450 °C on both process scales. It can be seen that the formation of gases is much more pronounced on a pilot scale, with a product yield of 47.84 wt.% in the experiment performed using 350 °C. As it was explored in Section 3.1, conducting PMMA depolymerization at 350 °C does not present excess heat to depolymerize the complete chain of the polymer, and the reaction terminates, favoring the formation of char through aromatization and condensation reactions. The pyrolysis of the generated char and liquid product (mainly MMA) at this low temperature favors the formation of gases, and its product yield increases. This effect was largely increased in the case of the pilot reactor, considering that the heat supplied by the LPG burner is controlled by a thermocouple located at the bottom center of the reactor. The increased length of the bed of PMMA particles compared with the lab-scale glass reactor associated with the poor heat conductivity of PMMA generates a temperature gradient between the heated rims of the cylindrical reactor and its center, where the thermocouple is located. Possibly, the combination of low temperature of depolymerization and slow heating rate favored the charring of PMMA waste over depolymerization. Even when considering a higher temperature of 450 °C to achieve random-chain scission of the polymer chain, the slow heating rate through the fixed bed is sufficient to generate a large quantity of char (17.5 wt.%). In the case of the lab scale, only a negligible quantity of char was obtained, since the effects of heat transfer can be minimized in such a small reactor. Strangely, the amount of gases decreased compared with the pilot-scale experiments. Since we did not analyze gas composition, we suspect this to be due to the fact that the 350 °C experiment was conducted for a much longer time than what was needed. The difference in reaction times is only 20 min, but considerably higher weight was used for 450 °C experiment (20 Kg), suggesting that the reaction could be conducted further in the case of 450 °C experiment, which could mean a higher yield of gases and a lower amount of char.

## 4. Discussion

In this work we detailed for the first time an accurate analysis of the semi-batch depolymerization process of EGDMA-crosslinked PMMA dental waste considering the influence of reaction time, kinetics and chemical composition variation at a high temperature of pyrolysis. The process is analyzed on different process scales, and the main problem associated with the scaling of fixed-bed semi-batch reactors is discussed. Through thermal analysis, we characterized and categorized the type of waste according to its decomposition profile and verify that crosslinking increases the thermal stability of the polymer and reduces its degradation at low temperatures, favoring main-chain random scission mechanism while suppressing the formation of H-H linkages in the polymer, causing it to degrade at low temperatures. In past studies, we did not observe this influence on the mechanism of thermal degradation [24,27]; in fact, we could verify the conclusions obtained by Da Ros et al. [35], who modeled the chemical recycling of EGDMA-crosslinked PMMA. Thermogravimetry analysis proves once more to be a valuable tool in the analysis of pyrolysis reactions and mechanisms, and the study of thermal degradation of determined feedstock is paramount to the design of pyrolysis reactors. We expect that further studies of thermogravimetry profiles of plastic waste could be used to infer characteristics such as the overall polymerization mechanism; i.e., we could use thermal analysis to obtain valuable information about waste, making it easier to process it in a hypothetical recycling factory.

Semi-batch reactors are cheap and easy to construct, but their correct operation depends upon considerable knowledge of the process in question due to its transient nature: the chemical composition of feed and vapors is ever-changing with the reaction time. In some processes, such as PMMA depolymerization, a semi-stationary state is obtained due to the depropagation of the polymeric chain into the monomer MMA. Our results showed that the liquid condensate of PMMA depolymerization is over 90% composed of it, but this composition is influenced by the reaction time, the temperature and, if the dimensions of the bed of polymer particles is considerable, the heating rate. A large bed of polymer particles increases temperature gradients between the rim and center of the reactor, overheating a part of the polymer bed and further reacting the diffusing formed MMA out of the bed, forming gaseous products. This observation is in accord with other researchers, who also identified condensate losses and increased gas yields for high-temperature depolymerization on a pilot scale [24,27,35]. The results show that it is possible to obtain high liquid-phase yields while maintaining high composition of MMA using semi-batch reactors with low processing temperatures.

This work further elaborates on the formation of char and secondary products of PMMA depolymerization at temperatures of 450 and 475 °C. While most plastics tend to thermally decompose forming almost no char, the mechanism of PMMA depolymerization clearly shows that depolymerization can be terminated before all the polymeric chain is consumed [47] and that, in a competitive manner, there are char formation reactions due to condensation, aromatization and hydrogen abstraction events of the formed depolymerization products, producing aromatic hydrocarbons, identified in the liquid phase of experiments at higher temperatures. This formed char cracks at higher temperatures, and the experiment at 425 °C produced the highest amount of char, but the liquid phase was composed only of MMA. Higher temperatures reduced formed char yields but increased the presence of aromatic hydrocarbons in the liquid phase, indicating that the formed char further cracked into these products. Higher temperatures improve the extent of cracking and depolymerization but can also pollute the liquid phase with undesired products. If one desires to maximize the recovery of MMA, one should aim to use the lowest possible temperature with low heating rates for fixed-bed reactors. High heating rates and high final temperatures would provoke low MMA recovery due to increased gaseous-phase yields (reducing liquid-phase yields) and lower purity of the liquid phase (lower MMA content). By conducting reactions at low heating rates and final temperatures, higher char yields are obtained (not that bad considering that it can be used for heating) as well as higher MMA recovery.

## Figures and Tables

**Table 1:** Main works considering semi-batch PMMA depolymerization process.

PMMA Type	Process Scale	T (°C)	Liquid Yield (wt.%)	% MMA	Investigation	Main Results	Reference
Model PMMA (PM = 350,000 g/mol)	Lab (1.5 g)	450	99.3	99.0	Polymerization of recycled monomer.	Contaminants in liquid phase slow down polymerization, and purification by fractional distillation is necessary.	[22]
Commercial PMMA	Lab (1.5 g)	450	98.1	96.8			
Homopolymer PMMA	Lab (30 g)	400	90.0	92.8	Process description and characterization of recycled polymer including test on a technical scale.	Polymerization was possible after fractional distillation. On a technical scale, liquid-phase yields were lower due to poor heat flow and diffusion of products in reaction kinetics, more evidently shown on a larger scale.	[23]
Dental waste	Lab (30 g)	400	90.0	92.8			
Dental waste	Technical (2 L)	400	66.3	76.4			
Dental waste	Pilot (143 L)	405	48.7	98.4	Process description on a pilot scale.	With a larger fixed bed of polymer, the effect of poor heat transfer in product yields is more evident.	[24]
Artificial marble powder (PMMA)	Lab (50 g)	400	88.8	76.9	Process investigation of pyrolysis of filled PMMA.	Experiments showed liquid-phase yields increasing with temperature, but MMA % decreased. The process showed large variation with heating rate, demonstrating that semi-batch reactors are heavily dependent upon the residence time of vapors inside the reactor. It is important to note that the reactor presented large dimensions compared with weight of feed (i.d. = 150 mm and L = 600 mm).	[26]

**Table 2:** Product yields of lab-scale PMMA dental waste depolymerization (350–450 °C).

Process Parameter	350 °C	400 °C	450 °C
Feed weight (g)	30.13	40.03	40.14
Total reaction time (min)	60	70	70
Initial condensation temperature (°C)	230	215	216
Final reaction temperature (°C)	350	400	450
Liquid-phase yield (wt.%)	48.76	91.06	94.74
Solid-phase yield (wt.%)	38.83	6.49	0.68
Gas-phase yield (wt.%)	12.41	2.45	4.58

**Table 3:** Physical–chemical properties of the liquid phase of PMMA dental waste lab depolymerization.

Property	MMA	350 °C	400 °C	450 °C
Density (g/cm^3^)	0.94	0.94	0.95	0.95
Acid Value (mg KOH/g)	0	1.92	1.93	1.92
Refraction Index (-)	1.49	1.42	1.42	1.42
Kinematic Viscosity (mm^2^/s)	0.621	0.589	0.596	0.594

**Table 4:** Product yields of PMMA dental waste depolymerization at 425–475°C.

Process Parameter	425 °C	450 °C	475 °C
Feed weight (g)	750.23	623.75	580.38
Total reaction time (min)	120	120	110
Initial condensation temperature (°C)	119	109	75
Final reaction temperature (°C)	440	455	475
Liquid-phase yield (wt.%)	81.63	61.67	61.32
Solid-phase yield (wt.%)	9.44	2.43	2.81
Gas-phase yield (wt.%)	8.93	35.90	35.86

**Table 5:** Comparison between experiments performed at different process scales (laboratory and pilot) considering the same temperatures of depolymerization (350 and 450 °C).

Process Parameter	Lab Scale		Pilot Scale	
	350 °C	450 °C	350 °C	450 °C
Feed weight (g)	30.13	40.14	15000	20,000
Total reaction time (min)	60	70	155	175
Initial condensation temperature (°C)	230	216	165	165
Final reaction temperature (°C)	350	450	350	450
Liquid-phase yield (wt.%)	48.76	94.74	44.50	55.00
Solid-phase yield (wt.%)	38.83	0.68	7.66	17.50
Gas-phase yield (wt.%)	12.41	4.58	47.84	27.50

## Data Availability

The raw data supporting the conclusions of this article will be made available by the authors upon request.
